# Systematically Investigating the Qualities of Commercial Encapsulated and Industrial-Grade Bulk Fish Oils in the Chinese Market

**DOI:** 10.3390/foods14091623

**Published:** 2025-05-04

**Authors:** Qian Zhou, Lili Xu, Yanan Xu, Qianqian Xue, Changhu Xue, Xiaoming Jiang, Yunqi Wen

**Affiliations:** 1 College of Food Science and Engineering, Ocean University of China, 1299 Sansha Road, Qingdao 266400, China; 2 Institute of Agro-Food Science and Technology, Shandong Academy of Agricultural Sciences, 202 Gongye North Road, Jinan 250100, China; 3 Qingdao Institute of Marine Bioresources for Nutrition & Health Innovation, Qingdao 266109, China

**Keywords:** fish oil, oxidation product, sensory evaluation, metal element, color, fatty acid composition

## Abstract

Fish oil is one of the most popular dietary nutritional supplements. Reports on the qualities of fish oils from Chinese markets are scarce, although the consumption of fish oil products in China is huge and increasing. This study systematically investigated the qualities of commercial encapsulated fish oils (CFs) and bulk fish oils (BFs) from Chinese markets, including oxidative level, sensory quality, color, metal element content, and unsaturated fatty acid content. Significant quality variations were observed both among individual CFs and between BFs: 65.2% of CFs (excluding one flavored sample) and one BF sample met China’s Grade II fish oil oxidation product standards; 80.8% of CFs and three BFs were within regulatory limits for heavy metal contamination. A distinct fishy odor was detected in four CFs and one BF sample, while a pronounced rancid odor was observed in one CF sample. The EPA contents in 64% of CFs and DHA contents in 48% of CFs met their labeled claims. Furthermore, these five quality parameter categories demonstrated non-significant intercorrelations, with the fish oil unit price being independent of quality. These findings indicated that most BFs require refinement, and CFs require implementation of low-temperature dark storage/transportation protocols. This study provided comprehensive quality benchmarks for fish oil production and marketing.

## 1. Introduction

Docosahexaenoic acid (DHA) and eicosapentaenoic acid (EPA) supplementations have been proven, in numerous clinical studies, to occupy promising effects, such as lowering inflammation and cardiovascular disease risk, improving cognition parameters of offspring, etc. [1,2,3]. Dietary supplementation is considered the most cost-effective approach to intake EPA and DHA, and the recommendation by FAO/WHO for total n-3 PUFAs was 1.4–25 g/day, and for EPA + DHA, it was 140–600 mg/day. Meanwhile, the American Heart Association recommends a daily dose of 1000 mg of EPA and DHA for people suffering from coronary heart disease (http://www.heart.org/HEARTORG/ (accessed on 1 March 2025); [4]. Fish oil is considered as one of the major natural sources of these two long-chain ω-3 PUFAs, and it can be ingested by the human body either orally through encapsulated fish oil supplements or via dietary incorporation into functional food products [5,6]. According to the information from the Ministry of Agriculture and Rural Affairs of the People’s Republic of China, the output of fish oil products in China was about 53.2 thousand tons in 2020, but the quantity demanded was about 90.5 thousand tons. EPA and DHA are highly susceptible to oxidation. The oxidation of fish oil leads the generation of undesirable hydroperoxide, odor, etc., and decreases the nutritional quality and safety of fish oil-containing foods [7,8]. Emerging toxicological data correlate lipid oxidation products with atherogenic potential, neurotoxic effects, etc. [9], while preclinical rodent studies substantiate dose-dependent hepatorenal toxicity, pro-atherosclerotic plaque formation, and growth axis disruption following sustained oxidized lipid intake [10,11,12].

Fish oil has been consumed as the daily supplement of ω-3 PUFAs for many years. It is needed to strictly monitor and control the oxidation product levels of fish oil. According to the international guidelines for the oxidation levels of fish oil supplements, the supplements should have a peroxide value (POV) of less than 2.5 mmol/kg, anisidine value (AnV) of less than 20, and Totox value (2POV + AnV) of less than 26 [13]. The Therapeutic Goods Administration (TGA) of Australia stipulates the POV and AnV of fish oil supplements should not exceed 5 mmol/kg and 30, respectively (https://www.tga.gov.au/compositional-guideline/fish-oil-natural (accessed on 1 March 2025)). The Chinese government also enacted a national standard for fish oil [14], classifying fish oils into two grades according to their qualities. Specifically, the first grade of fish oil should have a POV of less than 2.5 mmol/kg and an AnV of less than 20, and the second grade of fish oil should have a POV of less than 5 mmol/kg and an AnV of less than 25. Heller et al. [9] analyzed the POV and AnV of 26 fish oil supplements available in pharmacies in Australia. According to international guidelines, 38% of fish oils exceeded the limitation for POV, and 25% of samples exceeded the limitation of AnV, while 33% of samples exceeded the limitation of Totox value. Ritter et al. [15] assessed the qualities of the 16 most popular fish oil supplements from American markets, revealing that a quarter exceeded the international guidelines for POV. To date, the oxidative product levels of commercially available fish oil within the Chinese market have not undergone comprehensive systematic investigation.

The EPA and DHA contents were important parameters reflecting the quality of fish oil supplements. Kleiner et al. [16] analyzed the EPA and DHA contents of commercial fish, krill, and algal oil supplements and then compared the tested amount to the tagged content indicated on the label by the manufacturer (i.e., label amount), revealing that the tested amounts in over 70% of samples were unsuccessful in meeting the label amounts. Ritter et al. [15] reported that among 16 of the top-selling fish oil supplements from American markets, over half of these samples did not accord with their label claims for EPA and DHA. The compliance between label claims and analytically determined concentrations of EPA and DHA in commercially available fish oil distributed through the Chinese market remains a knowledge gap until now.

The sensory quality of fish oil, especially fishy odor, is an important factor impeding the application of fish oil in foods as a nutritious supplementary [1,6]. Encapsulation technologies are usually employed in fish oil formulations intending for direct ingestion. However, postprandial regurgitation-induced release of residual organoleptic impurities—particularly fishy odor—compromises consumer acceptability. In addition, physical encapsulation of fish oil can moderately improve its oxidative stability, which is widely used in the storage and transportation of fish oil products [17]. The levels of metal elements and color are two other important indicators reflecting fish oil quality. Therefore, twenty-six commercial encapsulated fish oils (CFs) and six industrial-grade bulk fish oils (BFs) in the Chinese market were sampled and analyzed for their oxidation product levels, sensory odor, metal element contents, color, and fatty acid compositions. In addition, the relationship between different quality indicators of fish oil was explored. This study is the first to integrate multiple quality parameters of commercially available fish oil for holistic assessment and systematically compare encapsulated and bulk fish oils. This systematic investigation could provide data support for the quality control of fish oil products in the circulation process of raw material storage, cold chain transportation, and terminal retail.

## 2. Materials and Methods

### 2.1. Standards and Reagents

Twenty-six CFs and six BFs were purchased from three commonly used shopping websites in China according to the sale ranking of fish oil. The fish oils were confirmed to be within the shelf life by label. For obtaining liquid fish oil, the capsule was punctured, and the oil was pressed into a 15 mL brown glass bottle. The encapsulating materials of all samples mainly consisted of gelatin according to the label. The obtained oil was blanketed with nitrogen before storage. All fish oil samples were stored at −20 °C until analysis. Given the susceptibility of fish oil to oxidation, the exposure time of fish oil to air during analytical experiments should be strictly limited to no more than 10 min. In addition, encapsulated fish oils and bulk fish oils were, respectively, numbered as CF1-CF26 and BF1-BF6. A Na_2_S_2_O_3_ solution (0.100 mol/L) for peroxide value determination and *p*-anisidine were purchased from Shanghai Yuanye Biotechnology Co., Ltd. (Shanghai, China). Deionized water was produced using a Millipore Milli-Q water purification system (Burlington, MA, USA). All other reagents were of analytical grade and obtained from Sinopharm Chemical Reagent Co., Ltd. (Qingdao, China). A fatty acid methyl ester (FAME) blend of 37 components was provided by ANPEL Laboratory Technologies Inc. (Shanghai, China).

### 2.2. Peroxide Value (POV) and p-Anisidine Value (AnV)

The determinations of POV and AnV were carried out according to our previous study [18]. Briefly, about 2 g of the oil sample was weighed into an iodine flask. A total of 30 mL of mixed solvent and 1 mL of saturated potassium iodide solution were used successively. After the reaction, 100 mL of distilled water was added, and then the titration with 0.0100 mol/L Na_2_S_2_O_3_-solution was performed. In total, 3 mL of 0.2% starch solution was used to visualize the end-point where the solution turned colorless.

The POV (mmol/kg) is calculated according to the following equation:

$$POV=\frac{(V-V0)\times c\times 1000}{2\times m}$$

V: the volume of Na_2_S_2_O_3_ solution consumed by the oil sample (mL); V0: the volume of Na_2_S_2_O_3_ solution consumed in the blank experiment (mL); c: the concentration of Na_2_S_2_O_3_ solution (0.0100 mol/L); m: oil sample weight (g).

The AnV was determined by recording the absorbance values using a Shimadzu UV-1700 spectrophotometer (Kyoto, Japan). This method utilized the chromogenic reaction between *p*-anisidine and a- and b- un-saturated aldehydes (primary 2-alkenes) in oil under the condition of acetic acid. The absorption of the solution was determined at 350 nm.

The AnV is calculated according to the following equation:

$$AnV=\frac{(1.2As-Ab)\times 25}{m}$$

As: the absorbance of the mixture of oil and isooctane, using isooctane as a blank control; Ab: after the chromogenic reaction, the absorbance of the mixture, using a mixture of isooctane and *p*-anisidine solution as a blank control; m: oil sample weight (g).

### 2.3. Sensory Assessment

The odor of fish oil samples was evaluated according to Wen et al. [18] with some modifications. The panelists were recruited from Ocean University of China (Qingdao, China), constituting by ten persons (8 female/2 male; aged between 22 and 45 years). The flavor vocabularies and attributes of fish oils were confirmed as fishy, frying, grassy, metallic and rancid after panelist discussion. Additionally, for accurately identifying the odor of fish oil samples, the definitions and references for these odor attributes were provided and prepared (Table S1). The odor training for the evaluation group was necessary and conducted in sensory laboratory (room temperature, mild light, and isolated compartments) for 10 days before the experiments using corresponding references. The odor intensity was assessed on a 0–10 point scale with 0.5 steps and represented by the following scores: 0—not perceptible, 2—very slightly perceptible, 4—slightly perceptible, 6—considerably perceptible, 8—strongly perceptible, and 10—very strongly perceptible. It should be mentioned that the panelists needed to refresh their noses with clean air for at least 15 min between the analysis of two samples to recover their sensory sensitivity. It is noteworthy that the samples of CF2, CF7, and CF10 presented a light lemon scent, which is because they contained an additional odor additive in the fish oil.

### 2.4. Metal Ion Determination

The fish oil was digested using a microwave digestion instrument (XT-Mul, Xintuo Analytical Instruments, Shanghai, China). The digestive solution was sent to ICP-MS (Agilent, Santa Clara, CA, USA) for the metal ion determination. The detailed experimental conditions were referred to in our previous study [19].

### 2.5. Color Measurements

The color of fish oil samples, including lightness (L), redness (a), and yellowness (b), were measured using a colorimeter (ColorQuest XE, Hunter Associates Laboratory Inc., Reston, VA, USA) with the standard observer of CIE 1964, the illuminant of D65, and 6.3 mm of aperture. The standard L, a, and b values were calibrated through a whiteboard and blackboard. The detailed operation process was conducted according to our previous study [20].

### 2.6. Fatty Acid Composition Analysis

The preparation of fatty acid methyl esters (FAMEs) and the detection through GC-MS (Agilent 7890a chromatograph coupled with an HP-5MS capillary column (30 m × 0.25 mm × 0.20 μm)) were conducted according to Xu et al. [3], with some modifications. About 20 mg of the sample was weighed into a tube, and 10% H_2_SO_4_ in methanol was added. Then, the tube was filled with nitrogen and heated at 90 °C for 90 min applying shaking every 20 min. After cooling, 1 mL of n-hexane was added to extract the FAMEs.

### 2.7. Statistical Analysis

All analyses of fish oil samples were performed in triplicate, and results were provided as mean ± standard deviation (n = 3). SPSS 20.0 software (SPSS Inc, Chicago, IL, USA) was applied in the statistical analysis of experimental data and the mean values were compared through one-way ANOVA, followed by Dunnett’s test. The statistical tests were considered to be significant at *p*-values less than 0.05. Origin 2017 (Northampton, MA, USA) was used for figure drawing, principal component analysis (PCA), and hierarchical cluster analysis (HCA), aiming to discriminate the samples from different groups.

## 3. Results and Discussion

### 3.1. Oxidation Levels of Fish Oils

**Figure 1:**
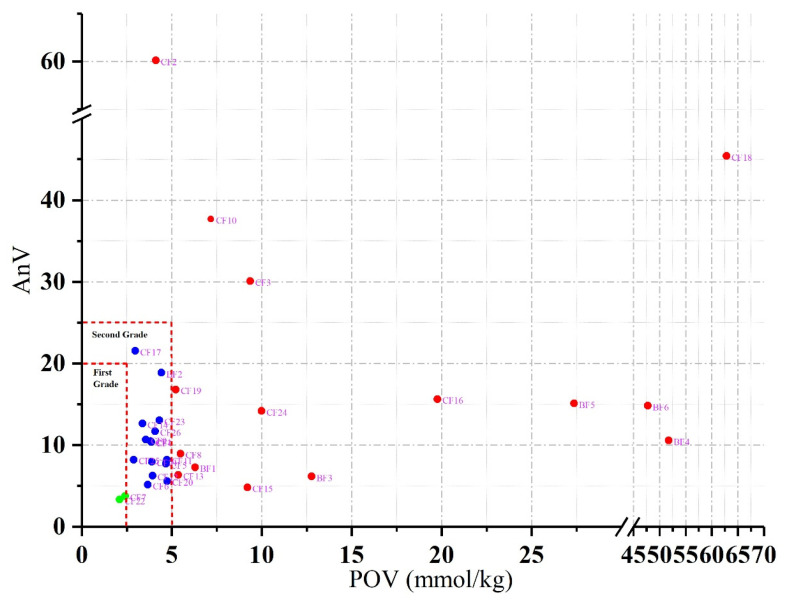
Peroxide values (POV) and *p*-anisidine values (AnV) of encapsulated fish oils (CFs) and bulk fish oils (BFs). Fish oils are classified into different grades according to their qualities (China aquatic industry standard SC/T 3502-2016 [14]).

The POV and AnV of CFs and BFs were analyzed and shown in Figure 1. As regards CFs, the POV ranged from 2.10 (CF22) to 62.82 (CF18) mmol/kg, with the average value of 7.60 mmol/kg. The AnV ranged from 3.34 (CF22) to 60.11 (CF2), with the average value of 14.88. In terms of BFs, BF2 and BF4, respectively, showed the minimum (4.42 mmol/kg) and maximum (51.74 mmol/kg) POVs. BF3 and BF2 showed the minimum (6.18) and maximum (18.86) AnVs. The Global Organization for EPA and DHA Omega 3 Voluntary Monograph propounds that the AnV determination is only valid for unflavored fish oils because the flavors could interfere with the AnV determination [13]. In this study, CF2, CF7, and CF10 were flavored fish oils with light lemon scent. Therefore, these three samples were excluded in the followed analysis involving AnV. According to China aquatic industry standard SC/T 3502-2016, only one sample (CF22) met the requirement of POV and AnV for first grade of fish oil, which accounted for 4.3% of 23 unflavored CFs. The POV and AnV of fourteen samples were within the limit values of the second grade of fish oil, which accounted for 60.9%. Eight other samples, accounting for 34.8%, showed high degrees of oxidation. With respect to BFs, only one sample (BF2) met the requirement of POV and AnV for the second grade of fish oil. The other five BFs showed a high degree of oxidation.

All encapsulated fish oil supplements marketed in New Zealand were measured for their POV and AnV, revealing that 83% of supplements exceeded the international guideline for POV, and 25% exceeded the AnV threshold [21]. Based on the oxidation data from a large third-party database, De Boer et al. [22] reported that 13.9% of fish oil exceeded the international guideline for POV (2.5 mmol/kg), and 2.2% exceeded the 5 mmol/kg. For AnV, 6.1% of samples exceeded 20, and 3.8% exceeded 30. According to the report by Bannenberg et al. [23], the ratio of POV/AnV was used to evaluate the oxidation time until the execution of the determination of POV and AnV. When the ratio is greater than 1, it means that the oil oxidation has occurred recently. As a result, only the ratio of CF15 was greater than 1, meaning that most of the selected fish oil samples had occurred with oxidation for a certain time. The oxidation of fish oil can deepen its color and deteriorate the sensory quality, which is influenced by various factors, such as the composition of unsaturated fatty acid, the form of fish oil product, temperature, etc. [24]. It was necessary to take measures to reduce the occurrence of oxidation during manufacture and especially transportation and storage, for example, storing fish oil in a low temperature and a dark environment, but which was not actually common in the marketing process.

### 3.2. Sensory Qualities of Fish Oils

**Figure 2:**
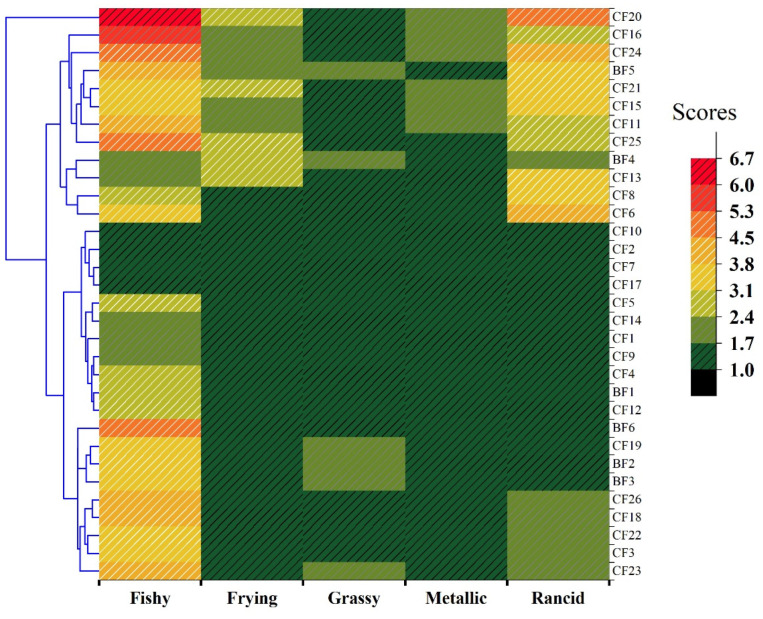
Heatmap of the sensory scores during the sensory evaluation of encapsulated fish oils (CFs) and bulk fish oils (BFs). The intensity of each odor attribute was evaluated on a 0–10 point scale with 0.5 steps. The flavor intensity represented by the score was 0—not perceptible, 2—very slightly perceptible, 4—slightly perceptible, 6—considerably perceptible, 8—strongly perceptible, and 10—very strongly perceptible.

Fishy, frying, grassy, metallic, and rancid were selected to profile the odor of fish oils. As shown in Figure 2, the fishy scores of fish oils ranged from 1.7 to 6.7 for CFs with the mean of 3.4 and ranged from 2.3 to 4.7 for BFs with the mean of 3.5, indicating that fishy odor presented a large difference between these fish oils. The rancid score also showed a large difference between fish oils. The rancid scores of CFs had a range of 1.0–4.7, with the average of 2.3, and the range was 1.0–3.3 for BFs, with the average of 1.7. The score ranges of frying, grassy, and metallic for CFs were 1.0–2.7, 1.2–2.0, and 1.0–2.3, respectively, and for BFs were 1.0–2.7, 1.3–2.0, and 1.0–1.7, respectively. The fish oil samples showed unsignificant difference in these three odors. According to the clustering analysis, the distance between CF2, CF7, CF10, and CF17 was close, meaning that their odor profiles were similar, and all of them presented very slightly perceptible odor. Considering CF2, CF7, and CF10, which contain a light lemon scent, the original odors of fish oils could be covered up to some extent. It was obvious in Figure 2 that CF20 had high scores for both fishy (6.7) and rancid (4.7), i.e., the emission of considerably perceptible or unacceptable smells from CF20. CF17 had low scores in the five odors evaluated. However, the AnV of CF17 was higher than that of CF20, which might be due to their difference in oil-refining processes. Song et al. [25] conducted seven deodorization methods during fish oil refining. Green tea polyphenol (GTP) treatment presented the best removal effect of the primary oxidation product, but it had a lower removal effect of fishy odor than that of zeolites and diatomite treatment methods. Meanwhile, these seven methods showed differences in the removal of the five odors and volatile components of fish oils. Therefore, different deodorization methods could have differential influence on the oxidation product content and fish oil odor.

### 3.3. Metal Element Levels

**Figure 3:**
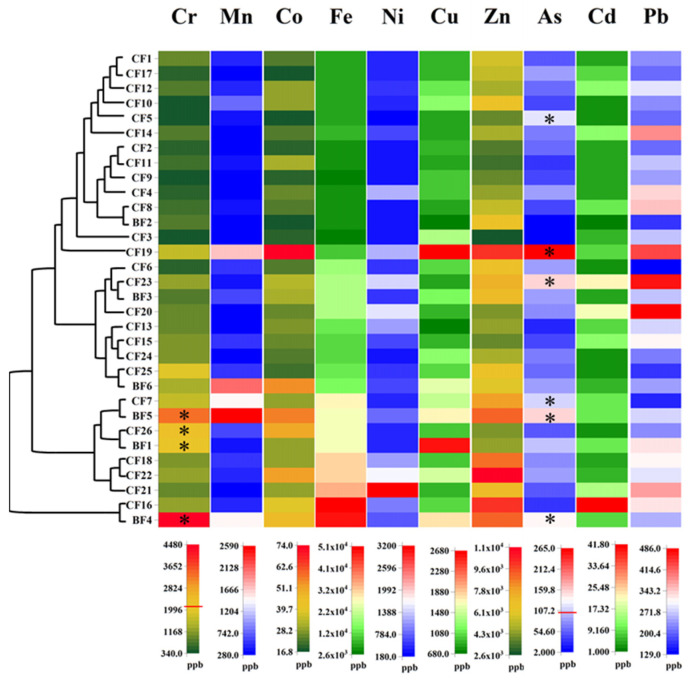
The distribution and clustering analysis of metal contents in encapsulated fish oils (CFs) and bulk fish oils (BFs). As the contents of different metal elements varied greatly in oils, each metal element had its corresponding content scale plate. The red line marked in the scale plate of Cr and As indicated their limited contents in seafood according to the Chinese food safety standard GB2762-2017. * Sample containing the metal element content exceeding the limitation.

Ten kinds of metal elements, including Cr, Mn, Co, Fe, Ni, Cu, Zn, As, Cd, and Pb, were measured for their contents in fish oils. As shown in Figure 3, the content range of Cr in CFs was 345.8–2299.1 μg/kg, with the average of 951.4 μg/kg, and in BFs was 771.0–4478.4, with the average of 2147.7 μg/kg. As for the other nine metal elements, the content ranges and corresponding average contents were shown in Table 1. The analyzed fish oils exhibited considerable variability in metallic composition, as evidenced by broad concentration ranges observed across all measured metal elements. Transition metal elements, including Cr, Mn, Co, Fe, Ni, Cu, Zn, and Cd, had high average contents, except Co and Cd. Heavy metal elements (As and Pb) had relatively low contents. The Chinese food safety standard [26] restricts the maximum contents of Cr, As, Cd, and Pb in seafoods with the threshold contents of 2000, 100, 300, and 1000 μg/kg, respectively. According to the standard, the Cr contents of one CF (CF26) and three BFs (BF5, BF1, and BF4) exceeded the limited concentration (Figure 3), resulting in a 3.8% over-limit ratio for CFs and 50% for BFs (Table 1). With regard to As, its contents in four CFs and two BFs exceeded the limitation, resulting in a 15.4% over-limit ratio for CFs and 33.3% for BFs. In summary, BFs had higher risk of the excess of As and Pb than that of CFs. Figure 3 also presents that CF1, CF17, CF12, and CF10 were classified into one cluster, and CF2, CF11, CF9, CF4, CF8, and BF2 were classified into another cluster, both of which showed relatively low metal element contents.

Wang et al. [19] analyzed the contents of metal elements in decolorized fish oil, reporting that the Cr, Mn, Fe, Cu, Zn, As, Cd, and Pb contents were 49.6, 91.2, 2178.9, 221.7, 233.7, 17.8, not found, and 27.0 μg/kg, respectively, all of which were lower than the average contents of corresponding metal elements in our study. The top-selling fish oil dietary supplements in the Turkey markets were evaluated of their heavy metal contents, showing that As was in the content range of 500–4190 µg/kg, and Cd was only found in one sample, with the content of 140 µg/kg, Fe in the range of 320–15,700 µg/kg, and Pb in the range of not detected—1010 µg/kg [27]. The preparation and refining process of fish oil had a great influence on the contents of metal elements. Different oil extraction methods were compared for obtaining the extracted fish oil with the least contamination. Four fish oils produced by Soxhlet extraction, enzymatic extraction, wet reduction, and supercritical fluid extraction showed large differences in the contents of heavy metal elements, and the supercritical fluid extraction was the best extraction method in reducing the heavy metal elements [28].

### 3.4. Color

**Figure 4:**
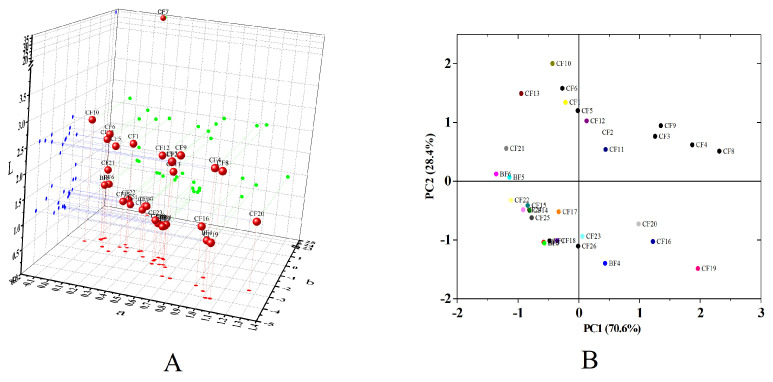
(**A**) The colorimetric results of encapsulated fish oils (CFs) and bulk fish oils (BFs), including lightness (L), redness (a), and yellowness (b). (**B**) The PCA of the colorimetric results of CFs and BFs except CF7.

The color of oil was related to its refining process and could reflect the quality of oil to a certain extent. CFs and BFs were photographed and are shown Figure S1. CF7 had relatively deep color, which might be caused by the additional ingredient emitting the lemon smell. The color of fish oils was also measured using a colorimeter (Figure 4A). L value indicated the brightness of the sample. Meanwhile, +a, −a, +b, and −b, respectively, indicated the levels of red, green, yellow, and blue values [29]. CF7 had the L, a, and b values of 34.7, 0.2, and 24.4, respectively, locating away from most of fish oil samples in the coordinate system. The ranges of L, a, and b for the other fish oil samples were 0.7–2.6, 0.1–1.3, and (−4.0)–(−0.5), respectively. The difference in color between samples was analyzed with a PCA model. As shown in Figure 4B, the color values of some samples showed the statistical difference, for example, CF10 and CF19, which could also be confirmed in Figure S1. The type of adsorbent used in the decolorization of fish oil, adsorbent content, process temperature, and time could affect the final color of decolorized fish oil. In addition, during the extraction of fish oil, different preparation methods also showed discriminative influence on the color of obtained fish oil [19,30,31]. It should be mentioned that capsule wall properties influenced fish oil color assessment, with significant variations in opacity and rigidity observed during collecting oil samples from capsules in this study.

### 3.5. Fatty Acids Compositions

The fatty acid compositions of fish oils were determined and shown in Table 2. The contents of EPA, DHA, MUFA, and PUFA ranged from 59.2 to 442.9 mg/g with the average of 184.1 mg/g, from 77.7 to 519.2 mg/g with the average of 154.0 mg/g, from 27.3 to 486.3 mg/g with the average of 288.2 mg/g, and from 185.9 to 753.0 mg/g with the average of 412.5 mg/g, respectively, indicating the larger difference in fatty acid compositions between fish oil samples. The EPA and DHA contents of 15 commercial fish oil supplements from the Turkish marketplace were assessed. The content ranges of EPA and DHA were 3.51–20.51% and 3.28–52.42%, respectively [32]. The variances in fatty acids likely arise from source fish species heterogeneity during fish oil manufacturing. Moreover, some fish oils are concentrated fish oils which undergo enrichment protocols using natural precursors to achieve high-potency EPA/DHA formulations.

### 3.6. Comparation of Label Amount and Measured Content of Fatty Acids

The measured amounts of EPA and DHA were compared with the label amount. The content gap between the test content and label content was calculated and expressed as PCG (percentage of content gap) in Table 2. The negative value of PCG meant that the test content was lower than the label content. For the PCGs of EPA and DHA, it varied from −19% to 49% and from −35% to 58%, respectively, indicating assayed EPA/DHA levels in some samples diverged significantly from label claims. Among 25 CFs samples (except CF7), nine and thirteen samples showed the negative values of PCGs for EPA and DHA, respectively, meaning that the EPA contents of 36% CFs and the DHA contents of 52% CFs failed to meet their corresponding label amounts. Only two samples demonstrated label-aligned DHA concentrations. As for BF samples, the EPA contents of two samples and the DHA contents of three samples could not reach the label amounts.

Albert et al. [21] analyzed the fatty acid compositions of 32 fish oil supplements in New Zealand, reporting that the concentrations of EPA and DHA in almost all samples were considerably lower than that of the label claims. Karsli [32] reported that as for 15 fish oil supplements in Turkey, the measured amounts of EPA were basically consistent with label amounts, but the DHA contents in some samples presented considerable difference with their labels. The phenomenon that the test amounts of EPA and DHA in fish oils were inconsistent with the label claims appeared in many studies. Considering the reasons, it may be that (1) the raw materials for the preparation of fish oils, i.e., fishes, when caught during different years or seasons, may be different in their fatty acid compositions; (2) fishes from different localities of growth may have differences in their fatty acid compositions; (3) the rearing methods of fishes may be changed, for example, the change in fodder; (4) the preparation methods of fish oil may be different; (5) manufacturers just want to reach the regulatory compliance threshold (80% of label claims); (6) fish oil undergoes a certain degree of oxidation during storage [6,15,16,30,33].

### 3.7. Evaluating the Unit Prices of EPA, DHA, MUFA, and PUFA

Based on the market price of fish oils and the test contents of fatty acids, the unit prices of EPA, DHA, MUFA, and PUFA were calculated (Table 2). The unit price of EPA varied from 1.0 to 80.3 ¥/g with the average of 13.4 ¥/g for CFs and from 0.5 to 3.5 ¥/g with the average of 1.1 ¥/g for BFs. The unit price of DHA was in the range of 1.7–48.1 ¥/g with the average of 11.1 ¥/g for CFs and of 0.5–1.2 ¥/g with the average of 0.9 ¥/g for BFs. Therefore, CFs’ EPA/DHA prices varied widely, while BFs’ remained stable. In addition, the average unit prices of EPA and DHA of CFs were higher than that of BFs. The unit price of MUFA showed the range of 0.6–138.0 ¥/g with the average of 9.5 ¥/g for CFs (except CF7) and of 0.2–2.0 ¥/g with the average of 0.6 ¥/g for BFs. The unit price of PUFA ranged from 0.5 to 18.7 ¥/g with the average of 4.1 ¥/g for CFs (except CF7) and from 0.2 to 0.7 ¥/g with the average of 0.4 ¥/g for BFs. Five samples showed the lower prices of MUFA than that of PUFA.

The selling price of fish oil is not only related to the contents of EPA and DHA, but also affected by many other factors, such as, the contents of micronutrients, brand, etc. For exploring the relationship between fatty acid content and the unit price of oil, linear regression analysis was performed. As shown in Table 3, *p* values of the linear regression models between the unit prices and EPA, MUFA, and PUFA were 0.47, 0.13, and 0.11, respectively, indicating these three regression models were not statistically significant (*p* > 0.05). The *p* value between the unit price and DHA content was less of 0.001, indicating the regression model was statistically significant with the R^2^ of 0.6. The positive coefficient of 0.018 indicated a direct correlation between DHA content and unit price, with higher concentrations associated with increased pricing. In addition, when using unit price to predict DHA content, the prediction accuracy was 60%.

The linear correlations between the unit price of fish oil and POV and AnV, were also evaluated. *p* values of the linear regression models between the unit price of oil and POV and AnV were 0.848 and 0.352, respectively, implying that these two regression models were statistically unsignificant (*p* > 0.05). Therefore, unsignificant linear correlation existed between the unit price of oil and POV and AnV; namely, the unit price of oil could not reflect the oxidation level of marketed fish oil.

### 3.8. Correlation Analysis Among Quality Indicators

**Figure 5:**
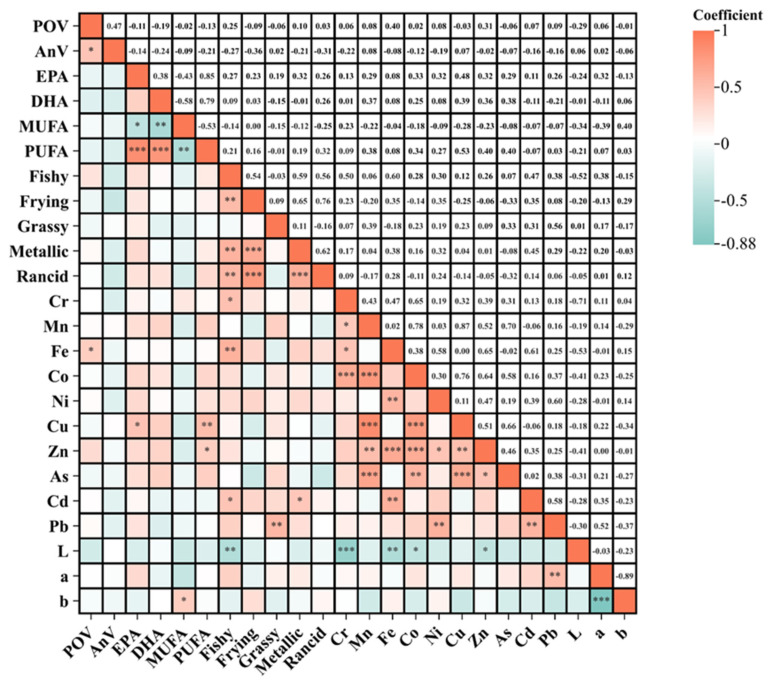
The correlation analyses among quality indicators. “*” stands for significantly different (*p* < 0.05), “**” stands for significantly different (*p* < 0.01), “***” stands for significantly different (*p* < 0.001).

POV and AnV serve as specific indicators for primary and secondary oxidation product quantification, respectively. Secondary oxidation products cause quality deterioration in fish oil, manifesting as intensified fishy odor, rancidity, and discoloration. The oxidation stability of fish oil was related to its fatty acid composition. The existence of transition metal ions in fish oil, acting as catalyst for oxidation reaction, could reduce the oxidation stability of fish oil [1,7,34]. Therefore, this study explored the correlation between oxidation product contents and sensory odor, color, metal element contents, and unsaturated fatty acid contents in CFs. As shown in Figure 5, POV only had a significant linear correlation with Fe (*p* < 0.05). Both POV and AnV showed negative linear correlation with PUFA content. The correlations between the unpleasant odor of fish oil and POV and AnV were weak. In other words, elevated PUFA content in fish oil did not inherently correlate with increased oxidation indices. Furthermore, oxidative status demonstrated non-linear relationships with off-odor intensity, defying conventional lipid oxidation chemistry assumptions. In addition, the oxidative levels of CFs showed uncorrelation with color and metal element contents.

Canonical correlation analysis is an effective analytical method to calculate the correlation between one set of variables and another set of variables and is applied to evaluate the correlation between oxidative levels and sensory odor, color, metal element contents, and unsaturated fatty acid contents. As shown in Table 4, the *p* values of four groups of canonical correlation analyses were all more than 0.05, indicating the coefficients were statistically meaningless. Therefore, the oxidation product levels of CFs showed uncorrelation with the sensory quality, color, metal element contents, and unsaturated fatty acid contents. The phenomenon might be attributed that fish oil demonstrated significant variations in oxidative status at the point of manufacture [35]. Although fish oils occurred with oxidation during the followed transportation, storage, and sales process, the amount of generated oxidation products was limited. During analyzing the oxidation product contents and fatty acid compositions in CFs, the measured levels were predominantly governed by the initial state established during the manufacturing phase. Under these circumstances, fish oil containing a high level of metal element or PUFA, may show a low level of oxidation products. Therefore, the levels of the five examined indices were not mutually inferable. However, it is important to note that increased oxidation levels observed in individual fish oil samples during transportation and storage were accompanied by a noticeable intensification of their unpleasant odor.

## 4. Conclusions

The qualities of commercially available CFs (n = 26) and BFs (n = 6) from China market were systematically investigated, including oxidative level, sensory quality, color, metal element content, and unsaturated fatty acid content. There was considerable variation in quality among individual CFs, as well as substantial differences observed between BFs. In general, 65.2% of CFs (excluding one flavored sample) met China’s Grade II fish oil oxidation standards (POV < 2.5 mmol/kg, AnV < 20), while only one BF sample satisfied these requirements. Regarding heavy metal contamination, 80.8% of CFs and three BF specimens did not exceed regulatory limits (Cr < 2000 μg/kg, As < 100 μg/kg). Sensory evaluation detected noticeable fishy odor in only four CFs and rancidity in one CF, with a single BF exhibiting pronounced fishy odor. Fatty acid composition analysis showed 64% and 48% of CFs met labeled EPA and DHA content claims, respectively.

The substandard qualities of most BFs necessitates refining processes. In addition, there was no significant correlation between the five categories of quality parameters studied, and the oxidative level served as a poor indicator for the levels of other four quality parameters. The unit price of fish oil showed no relationship with its quality. It was difficult for consumers to intuitively and accurately judge the quality of fish oil. This study provided comprehensive quality data for fish oil producers and sellers. Low temperature and dark condition during storage and transportation of fish oil should be promoted. Additionally, for consumers, the product unit price should not be the sole basis for purchasing high-quality fish oil products. Generally, product labels do not specify the need for cold storage. However, this study recommends storing fish oil products in refrigerator to slow down the generation of oxidation products.

## Figures and Tables

**Table 1:** The minimum, maximum, mean, limited content, and corresponding over-limit ratio of metal element in CFs and BFs.

Metal	CFs			BFs			Limited Content (μg/kg) *	Over-Limit Ratio (%)	
	Minimum (μg/kg) (Sample)	Maximum (μg/kg) (Sample)	Mean (μg/kg)	Minimum (μg/kg) (Sample)	Maximum (μg/kg) (Sample)	Mean (μg/kg)		CFs	BFs
Cr	345.8 (CF3)	2299.1 (CF26)	951.4	771.0 (BF2)	4478.4 (BF4)	2147.7	2000	3.8	50
Mn	296.2 (CF14)	1688.2 (CF19)	511.9	282.8 (BF2)	2582.3 (BF5)	1250.3	/	/	/
Fe	2769.0 (CF3)	50,979.7 (CF16)	14,900.1	4381.3 (BF2)	48,823.7 (BF4)	23,415.5	/	/	/
Co	16.9 (CF5)	74.0 (CF19)	29.3	18.7 (BF2)	55.1 (BF5)	39.0	/	/	/
Ni	189.4 (CF5)	3195.0 (CF21)	795.8	335.8 (BF2)	802.1 (BF5)	576.2	/	/	/
Cu	707.6 (CF13)	2670.9 (CF19)	1080.1	689.1 (BF2)	2553.6 (BF1)	1574.7	/	/	/
Zn	2584.7 (CF3)	11,306.8 (CF22)	5819.2	4587.6 (BF1)	9227.1 (BF5)	7080.9	/	/	/
As	2.7 (CF3)	264.7 (CF19)	72.2	6.1 (BF2)	158.0 (BF5)	95.4	100	15.4	33.3
Cd	2.9 (CF26)	41.6 (CF16)	9.2	1.1 (BF2)	10.0 (BF1)	6.4	300	0	0
Pb	129.9 (CF6)	485.5 (CF20)	284.3	165.1 (BF2)	328.5 (BF1)	256.2	1000	0	0

* The limited contents in seafood according to the Chinese food safety standard GB2762-2017 [26].

**Table 2:** The label claims of EPA and DHA, the test contents of EPA, DHA, MUFA, and PUFA, and the unit prices of fatty acids in CFs and BFs.

Samples	EPA (mg/g)				DHA (mg/g)				MUFA (mg/g)		PUFA (mg/g)		Price of Oil
	Label	Test	PCG *	Price **	Label	Test	PCG	Price	Test	Price	Test	Price	
CF1	183.00	173.10	−5.0%	6.82	120.00	94.90	−21%	12.43	282.53	4.18	331.31	3.56	1.18
CF2	180.00	165.00	−8.0%	1.94	120.00	101.45	−15%	3.15	259.41	1.23	314.99	1.02	0.32
CF3	178.00	160.70	−10%	2.87	103.00	103.12	0%	4.47	240.19	1.92	336.29	1.37	0.46
CF4	141.00	189.87	35%	1.14	94.00	116.33	24%	1.86	261.80	0.83	354.99	0.61	0.22
CF5	60.00	88.08	47%	18.92	60.00	94.68	58%	17.60	486.33	3.43	260.64	6.39	1.67
CF6	100.00	134.09	34%	80.27	480.00	519.15	8.0%	20.73	78.00	137.98	690.47	15.59	10.76
CF7	45 mg ^†^	170.82	\	36.67	150 mg ^†^	409.76	\	11.00	27.32	\	646.67	\	\
CF8	180.00	170.45	−5.0%	1.76	120.00	103.53	−14%	2.90	269.93	1.11	336.36	0.89	0.30
CF9	141.00	156.08	11%	0.99	94.00	88.85	−5.0%	1.74	247.36	0.63	325.30	0.48	0.16
CF10	180.00	146.47	−19%	2.01	120.00	94.32	−21%	3.13	287.61	1.03	315.73	0.93	0.30
CF11	150.00	156.59	4.0%	2.90	100.00	91.24	−9.0%	4.98	255.76	1.78	338.08	1.34	0.45
CF12	180.00	196.64	9.0%	1.98	125.00	117.63	−6.0%	3.32	327.59	1.19	366.35	1.06	0.39
CF13	180.00	177.41	−1.0%	4.07	120.00	120.06	0%	6.02	267.31	2.70	375.73	1.92	0.72
CF14	60.00	75.43	26%	45.96	100.00	96.60	−3.0%	35.89	447.42	7.75	272.67	12.71	3.47
CF15	180.00	203.59	13%	1.47	120.00	126.59	5.0%	2.37	412.47	0.73	417.69	0.72	0.30
CF16	180.00	171.24	−5.0%	9.64	120.00	104.55	−13%	15.78	258.96	6.37	287.25	5.74	1.65
CF17	300.00	350.11	17%	3.62	200.00	223.43	12%	5.67	279.98	4.52	663.94	1.91	1.27
CF18	180.00	153.04	−15%	10.21	120.00	77.73	−35%	20.10	285.30	5.48	358.22	4.36	1.56
CF19	300.00	382.64	28%	6.08	200.00	288.32	44%	8.06	194.65	11.94	753.04	3.09	2.33
CF20	460.00	442.87	−4.0%	2.91	180.00	172.54	−4.0%	7.48	136.48	9.46	682.75	1.89	1.29
CF21	192.80	212.99	10%	1.17	101.00	121.74	21%	2.05	378.50	0.66	441.78	0.57	0.25
CF22	320.00	364.16	14%	6.35	240.00	264.67	10%	8.73	240.95	9.59	738.43	3.13	2.31
CF23	60.00	60.79	1.0%	30.84	100.00	85.34	−15%	21.97	344.54	5.44	185.90	10.09	1.88
CF24	150.00	223.73	49%	6.70	100.00	134.51	35%	11.15	407.92	3.68	477.89	3.14	1.50
CF25	180.00	225.05	25%	4.40	120.00	146.60	22%	6.75	395.03	2.51	452.52	2.19	0.99
CF26	60.00	72.97	22%	57.10	100.00	86.66	−13%	48.08	388.84	10.72	222.80	18.70	4.17
BF1	150.00	168.18	12%	0.65	100.00	92.31	−8.0%	1.19	271.68	0.40	315.17	0.35	0.11
BF2	60.00	59.21	−1.0%	1.01	120.00	115.70	−4.0%	0.52	380.17	0.16	228.21	0.26	0.06
BF3	180.00	175.10	−3.0%	0.46	120.00	103.06	−14%	0.78	283.80	0.28	351.67	0.23	0.08
BF4	100.00	139.22	39%	3.45	400.00	419.86	5.0%	1.14	236.78	2.03	661.47	0.73	0.48
BF5	150.00	167.85	12%	0.54	100.00	109.90	10%	0.82	304.10	0.30	358.89	0.25	0.09
BF6	150.00	159.09	6.0%	0.57	100.00	102.26	2.0%	0.88	282.04	0.32	337.24	0.27	0.09

MUFA: Monounsaturated fatty acids. PUFA: Polyunsaturated fatty acids. CFs: Encapsulated fish oils. BFs: Bulk fish oils.* Percentage of content gap: PCG = (Test content − Label content) × 100%/Label content. ** The unit price using RMB was ¥/g. ^†^ The weight of EPA and DHA in each capsule. The label of CF7 unmarks the weight of capsule and the contents of EPA and DHA.

**Table 3:** Linear regression analyses between the unit price of oil and fatty acid contents and POV and AnV.

Linear Correlation	Price and EPA	Price and DHA	Price and MUFA	Price and PUFA	Price and POV	Price and AnV
R^2^	0.023	0.6	0.097	0.107	0.002	0.038
*p*	0.47	<0.001	0.13	0.11	0.848	0.352
Significance	N *	Y	N	N	N	N
Coefficient	/	0.018	/	/	/	/

* N indicates the regression model was not statistically significant. Y indicates the regression model was statistically significant.

**Table 4:** Canonical correlation analyses between G1 and G2, G3, G4, and G5.

Correlation	G1 and G2 *		G1 and G3		G1 and G4		G1 and G5	
	A1 **	A2	B1	B2	C1	C2	D1	D2
Coefficient	0.513	0.312	0.541	0.251	0.367	0.075	0.853	0.431
*p*	0.617	0.727	0.396	0.721	0.789	/	0.179	0.939
Significance	N ^†^	N	N	N	N	N	N	N

* G1 indicates Group 1, consisting of POV and AnV. Group 2 consisted of the scores of fishy, frying, grassy, metallic, and rancid. Group 3 consisted of the contents of EPA, DHA, MUFA, and PUFA. Group 4 consisted of the values of L, a, and b. Group 5 consisted of the contents of Cr, Mn, Co, Fe, Ni, Cu, Zn, As, Cd, and Pb. ** A1 and A2 are, respectively, first and second canonical variable for the canonical correlation analysis between G1 and G2. ^†^ N indicates the statistically meaningless coefficient.

## Data Availability

The original contributions presented in the study are included in the article, further inquiries can be directed to the corresponding authors.

## Supplementary Materials

The following supporting information can be downloaded at: https://www.mdpi.com/article/10.3390/foods14091623/s1, Figure S1: Photos of encapsulated fish oils (CFs) and bulk fish oils (BFs) placed in culture dish; Table S1: Descriptions, definitions and references for the odor attributes indicated by the panelists for fish oil.
