# Supplementary material for: Systematically Investigating the Qualities of Commercial Encapsulated and Industrial-Grade Bulk Fish Oils in the Chinese Market
Source: Foods. 2025 May 4;14(9):1623. doi: 10.3390/foods14091623 (PMC12071204; doi:10.3390/foods14091623)

Table S1 Descriptions, definitions and references for the odor attributes indicated by the panelists for fish oil.

Odor attribute	definitions	References
Fishy	an aromatic reminiscent of cod liver oil	cod liver oil diluted in good-quality soybean oil
Rancid	an aromatic reminiscent of odour or flavor of highly oxidized oils containing high amount of linolenic acid such as sunflower, cottonseed, or peanut	Good quality cottonseed oil aged for four days at 60 °C or until a peroxide value of approximately 5.0 is reached
Grassy	an aromatic reminiscent of the green character of mowed grass	Crude soybean oil from non-heat-treated soybeans diluted in good-quality soybean oil
Frying	an aromatic reminiscent of fried bread stick	Soybean oil that has been used to fry deep-fried dough sticks
Metallic	an aromatic associated with metal coins	0.01% ferrous sulfate diluted in distilled, filtered water

Figure S1 Photos of encapsulated fish oils (CFs) and bulk fish oils (BFs) placed in culture dish.

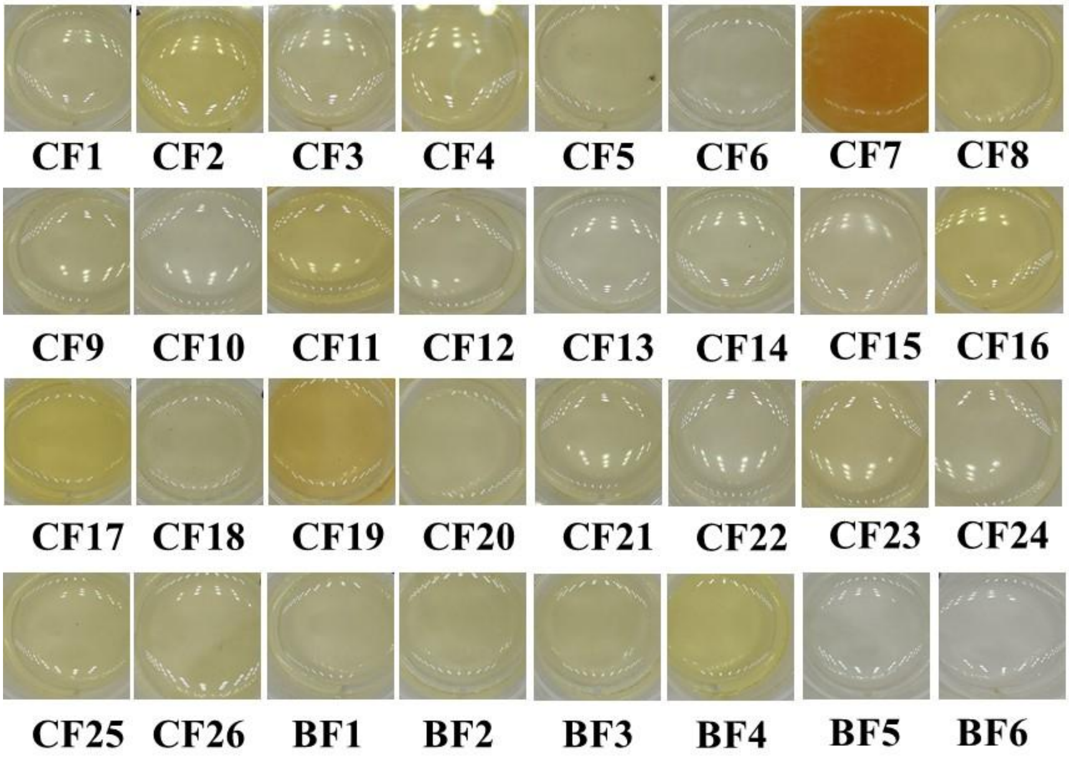

Supplement: Supplementary file 1 [file foods-14-01623-s001.zip › foods-3604772-supplementary.pdf]
